# A Method for Behavior Change Support by Controlling Psychological Effects on Walking Motivation Caused by Step Count Log Competition System

**DOI:** 10.3390/s21238016

**Published:** 2021-11-30

**Authors:** Kyosuke Futami, Tsutomu Terada, Masahiko Tsukamoto

**Affiliations:** 1 College of Information Science and Engineering, Ritsumeikan University, Kusatsu 525-8577, Japan; 2 Graduate School of Engineering, Kobe University, Kobe 657-8501, Japan; tsutomu@eedept.kobe-u.ac.jp (T.T.); tuka@kobe-u.ac.jp (M.T.)

**Keywords:** information presentation, competition information, sensor log, psychological effect, cognitive bias, behavior change, motivation, step counts, persuasive technology, nudge, human–computer interaction (HCI)

## Abstract

Systems presenting information that encourages competition by using rankings and scores (hereafter referred to as competition information) have become widespread to support behavioral change. However, users without high levels of motivation, such as behavior change support targets, do not necessarily benefit from competition information. In this study, we propose a method to control the psychological effects caused by competition information to support behavior change. We implemented a competition information presentation system using step counts logs to support increasing one’s daily steps. We designed two patterns of competition information considering psychological effects. One is likely to have good effects, using three mechanisms to easily obtain results corresponding to the effort, make closely matched rivals with similar abilities, and pay attention to a small number of rivals. The other is unlikely to have positive effects and may potentially even have negative ones, using a mechanism that brings about the opposite results of the former pattern. We evaluated 42 participants with low levels of motivation over six weeks. The results showed that the former information pattern increased participants’ step counts by about 1000 steps per day, and the latter information pattern did not lead to an increase. We confirmed the feasibility of the proposed method and discussed the possibility of the appropriate use and potential abuse of such techniques for manipulating motivation. Our study can be helpful in designing a competition information presentation system considering psychological effects.

## 1. Introduction

Methods for supporting behavioral change have been developed along with ubiquitous and wearable computing [1,2]. Small computer devices are suitable for such methods because they can monitor a user’s behavior and present information to intervene in users’ activities. These methods have been gaining attention in many areas to address many social issues, such as health risks due to lack of physical activity [3,4].

To support behavioral change, systems presenting information that encourages competition using rankings and scores (hereafter referred to as competition information) have become widespread and are used to encourage various behaviors (e.g., exercise and learning [5,6]). As competition information is known to cause psychological effects that unconsciously affect motivation [7,8,9,10,11,12,13], previous methods have aimed to use such effects for behavior change support. Such systems using competition information are expected to continue to be used for various behavior change supports.

Users without high levels of motivation, such as behavior change support target users, do not necessarily benefit from competition information. For example, if a user who increased the amount of effort they put in can achieve a result that reflects the user’s effort (e.g., a rank rises, a score difference from higher rank rivals becomes smaller), the user’s motivation is assumed to improve. Contrary to this, the user’s motivation is assumed to decrease if a result does not correspond to their effort. If there is a possibility that the effects the competition information presentation systems have on a user’s motivation can be good or bad depending on the information presentation factor, and that they can be intentionally manipulated, then this should be explained to users. Furthermore, based on this possibility, a competition information presentation system needs to be improved to be more effective for behavior change support. However, few previous studies have verified or discussed this point of view.

In this study, we propose a method to control the psychological effects of competition information for supporting behavior change. In addition, we verify examples of competition information that are good for supporting behavioral change and not good for it. We implemented a system using step counts logs to support increasing one’s daily steps. We designed two patterns of competition information. One is likely to have good effects, using three mechanisms to easily obtain results corresponding to the effort, make closely matched rivals with similar abilities, and pay attention to a small number of rivals. The other is difficult to have good effects or may have bad effects. Then, we evaluated our systems over six weeks, using 42 people who had low levels of motivation to increase step counts. Note that we defined the bad effects on motivation in this study as two effects: one that causes a decrease in motivation, and one that prevents an increase in motivation.

## 2. Related Work

### 2.1. Psychological Effects Caused by Competition

In this paper, we try to promote the good effects of competition information systems. We consider that the following three factors of competition information unconsciously influence the user’s mind.

We assumed that whether or not there is always a rival with a close step count in the competition information of step counts is related to whether or not the competition information can easily have a positive effect on motivation. The perception of the difference between others and oneself is known to affect motivation. To enhance the mind, small differences in terms of ability or performance with others are important [14] because only similar rivals become comparison targets [15,16]. For example, effort in a cognitive task increases when rivals are close in ability [7], comparison concerns became stronger in small groups with similar rivals [8,9], and academic ability increases with similar level rivals [17]. Motivation increases in a group including rivals at similar levels and declines in a group including rivals at very different levels [10]. A change in mind caused by such deference is rooted in the fundamental and universal desires of animals, such as self-assessment for comfortable survival [18], self-improvement by comparing with higher others [19], and self-elevation by comparing with lower others [20].

We assumed that the ease of obtaining results (e.g., rank fluctuation) corresponding to effort in the competition information is related to whether or not the competition information can easily positively affect motivation. The feedback for behavior is known to influence motivation and behavior change. Feedback works as a reward/punishment, which enables reinforcement learning and conditioning [21]. For example, a user’s effort is strengthened when feedback is a positive experience; conversely, his/her effort is weakened when feedback is a negative, unrewarded experience [21]. It is important to develop a mechanism to improve the perception that results can be obtained according to efforts [22]. In addition, feedback affects the mind by creating elements, such as achievement/reward experiences and perception of the current situation [1]. Achievement experiences lead to a positive mind, such as feelings of self-efficacy with regard to accomplishing a task [23], which increases the success rate, effort [24], and motivation [25].

We assumed that the number of rivals shown by the competition information is related to whether or not the competition information can easily positively affect motivation. The number of rivals presented affects motivation. Estimates of winning tend to be predicted as being higher when the number of rivals is smaller [11,12] and the degree of concern regarding comparing with others decreases as the number of rivals increases [13]. For example, the motivation and achievement levels of tasks become higher when there are only ten rather than 100 rivals [11]. In addition, academic test scores are higher when participants were in a small group rather than a big group [11]. Competitive attitudes increase as the number of rivals decreases [26] and as rivals are identified rather than unspecified [27]. The personality trait that causes this tendency is the degree of concern regarding social comparisons [11]. Similar phenomena in which the number of rivals presented affects the mind also occur in situations where one has to cooperate with others, such as the Ringelmann effect [28]. His/her position at ranking is also important in enhancing motivation. For example, a higher-ranked person has more motivation than a middle-ranked person [29].

### 2.2. Information Presentation Method to Control Psychological Effects

Recently, the importance of research focusing on unconscious phenomena such as psychological effects and cognitive biases caused by information presentation systems has been demonstrated [30]. These studies have been helpful for designing information presentation systems and demonstrated that manipulating the psychological effects of information presentation can provide effective support for users. Inspired by these, we focus on the psychological effects caused by competition information presentation systems that use walking logs.

Some methods aim to change behaviors and choices. Takeuchi et al. [31] proposed a method that encourages users to select healthy meals by presenting other people’s evaluations (e.g., likes) of a user’s meal content, posted on a system similar to an SNS (Social Networking Service). Shen et al. proposed a method for controlling tourist flow and reducing congestion by changing the tourist route customization screen and store rankings [32,33]. Futami et al. [34] proposed a method for encouraging the behavior making a train on time by showing the train schedule that is different from the actual one. For improving users’ thoughtfulness in posting a comment online, Menon et al. [35] proposed an interface design.

Some methods aim to improve concentration and productivity in work. Kim et al. [36] proposed using a work productivity log based on framing effects, and Ban et al. [37] proposed changing the time elapsed speed on a digital clock. Some methods aim to improve the quality of dialogue communication. Costa et al. [38] proposed a method for lowering psychological stress during discussions by modifying the voices of oneself and others. Suzuki et al. [39] proposed a method for improving the quality of collaborative work by changing the facial expressions of dialogue partners from the actual ones. Arakawa et al. [40] proposed a method for improving users’ concentration on video teaching material by detecting the decrease in their concentration with machine learning and modulating the video sound.

Some methods aim to transform the state and ability of the mind and body. Costa et al. [41,42,43] proposed a method to enable users to unconsciously improve the their cognitive performances and mental states by presenting false heart rate sensor information. To improve the user’s emotional and mental states, Yoshida et al. [44] proposed a feedback method that modifies the user’s facial expression to be more positive than the actual one. In addition, to improve the user’s ability to stay calm in terms of mind and body in tense moments in sports, Futami et al. [45] proposed a method that presents success conditioned sounds, and Tagami et al. [46] proposed a method to present a pseudo-success experience.

Some methods aim to change sensory perception. To manipulate the elapsed time sensation, there is a method that changes the frequency and timing of auditory stimuli from a speaker [47] and tactile stimuli from a wrist-worn device [48], a method that changes the movement speed of visual icons on an HMD (Head Mounted Display) [49]. To control the physical burden (e.g., subjective fatigue) when lifting objects, Futami et al. [48] proposed a method that modifies and presents the sensor values of muscle activity, and Ban et al. [50] proposed a method that changes the color of the object to be lifted with XR technology. Narumi et al. [51] proposed a method that controls users’ feelings of fullness by changing the visual size of food to eat. Adams et al. [30] proposed a method for changing the perception of the amount of food on the plate by changing the apparent size of the plate.

### 2.3. Promoting Physical Exercise Behavior

In this paper, we implement an information presentation system to promote an increase in step counts. Promoting physical exercise has been gaining attention because an increase in the amount of exercise is vital for mental and physical health, such as disease prevention [3,4]. For example, since even a simple increase in one’s number of steps is medically effective [52], simple logging with pedometers has been used [53,54,55]. However, many people do not regularly exercise. Therefore, many support methods have been proposed. Among them, feedback methods are often used for encouraging reflection and behavior conditioning (i.e., reinforcement learning) [21]. The feedback content varies, presenting things such as the values of achievement level [53,54,55], growth status in breeding (e.g., pets [56] and gardens [57,58] that grow according to user’s exercise), and symbols of reward/punishment and achievement/failure (e.g., icons such as facial emoticons [59], badges and awards [60], congratulatory messages and simple symbols [61]). Feedback methods with competition have been widely used, such as a system for providing feedback on competition progress with game scores [5] and large-scale municipal events of step-counting competitions. Other methods, such as setting appropriate goals [62] and persuading according to the stage of behavioral change [63], have also been used to increase physical activity [64,65]. Some methods were developed for specific ages (e.g., seniors [66]) and girls [67]. These previous studies show that promoting physical exercise is an important topic of behavior change. Our study outcome is expected to be useful for designing an exercise behavior supporting system.

## 3. Methods

**Figure 1:**
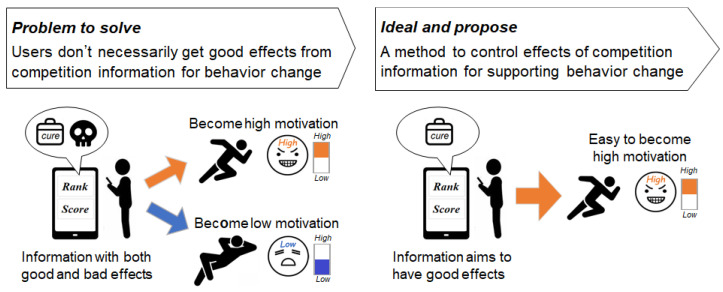
The effects of a competition information presentation system on behavioral motivation vary depending on the information pattern. This paper examines methods to promote good effects of competition information.

This section describes the system’s design for verifying the effect on motivation caused by a competition information presentation of the step log competition system. Figure 1 shows the problem and proposal of the subject of this study.

### 3.1. Target Behavior and Competition Game Rules

In this study, a step log competition system was selected as a case study. The rules of the game were a general competition of incremental steps. Specifically, the rules are as follows: Users compete with scores of incremental steps. The number of steps increases while walking and running. Information on the competition progress is updated and presented daily. We selected these for the following reasons: (1) Studying how to increase the number of steps taken by an individual is an important and popular area of research because such an increase is effective for improving health; (2) In most cases, the primary purpose of a competitive game of counting the number of steps is to support behavioral change instead of pure competition to determine a winner; (3) As such competition games are already general and widespread, our research outcomes can be used and socially implemented for various target behaviors.

### 3.2. Design of Method 1

Method 1 is a presentation method of a rank and score. Method 1 controls the following two elements.

**Figure 2:**
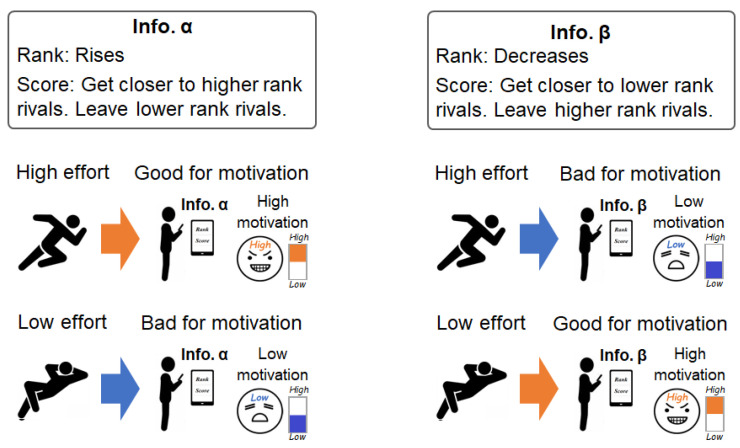
Method to control the effects of competition information on motivation by presenting results corresponding to the effort.
**Element 2: Degree of difference from rivals**.
  The perception of the difference between others and oneself influences motivation. The presence of a rival at the same level increases motivation, while the presence of only rivals with a large difference in level decreases motivation. When using the step log competition information presentation system, the difference in score between rivals is assumed to affect motivation. For example, when a user perceives a rival with a similar score, the user’s motivation is likely to increase. However, when a user perceives a situation with only rivals with a large score difference, improvements in motivation are likely to be hindered.
  Based on this assumption, Method 1 has a mechanism that manipulates score differences from rivals. Figure 3 shows an image of the change in motivation caused by this factor.

**Element 1: Results corresponding to the user’s effort**.
  The feedback for behavior influences motivation and behavior change. When using the step log competition information presentation system, the user’s motivation is assumed to be influenced by the ease of obtaining results according to their effort. For example, in an environment where positive results (e.g., a rank rises) are easily achieved when the amount of effort increases, it is assumed that motivation is likely to increase. On the other hand, it is assumed that improvements in motivation are likely to be hindered where positive results are difficult to obtain, even when increasing the amount of effort.
  Based on this assumption, Method 1 has a mechanism that manipulates the ease of obtaining results corresponding to the amount of effort. Figure 2 shows an image of the change in motivation caused by this factor. The results according to the amount of effort here highlight the following two points: (1) When the effort increases, the rank increases, the difference in scores between higher-rank rivals decreases, and the difference in score between lower-rank rivals increases. (2) When the effort decreases, results opposite to those of (1) are provided.

**Figure 3:**
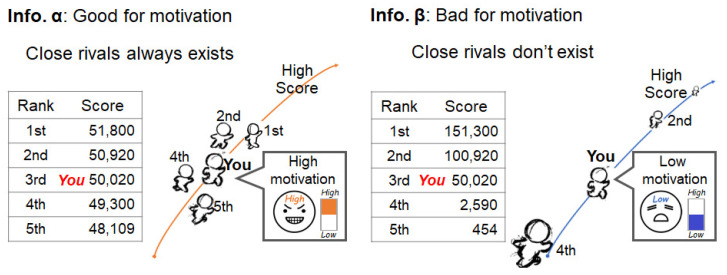
Method to control the effects of competition information on motivation by presenting the score difference compared to rival players.

#### Algorithm for Rank and Score

**Figure 4:**
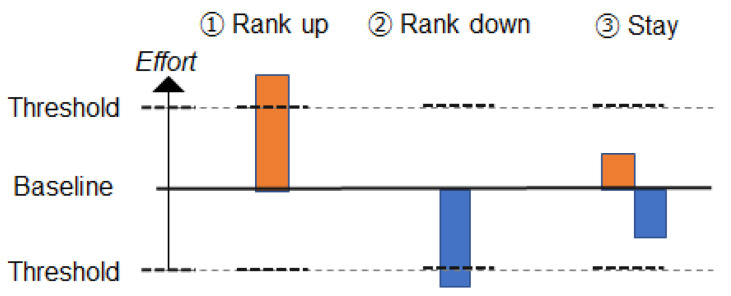
Rank fluctuation depending on the amount of effort.

**Figure 5:**
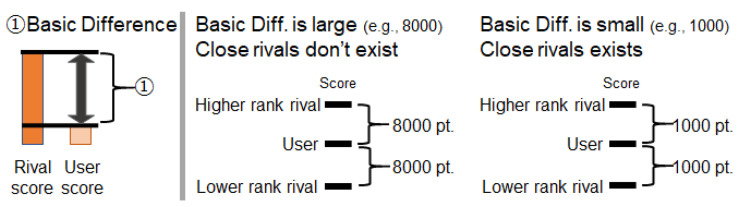
Example of how the score differences from rivals are adjusted with Basic Difference Factor.

**Figure 6:**
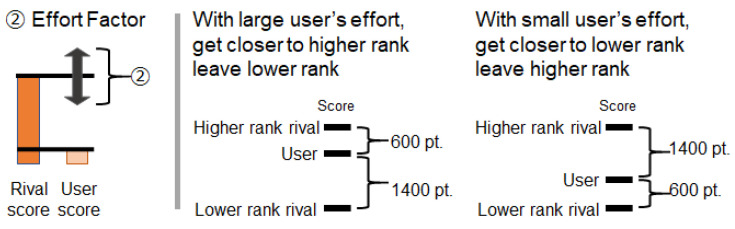
Example of how Effort Factor reflects user’s effort on the score difference from rivals.

The rank and score differences are manipulated as follows:

**About Rank**
  Rank fluctuation occurs as follows: A rank fluctuation occurs when the amount of the user’s effort exceeds the threshold for a rank fluctuation. Figure 4 shows an overview of this. Depending on the user’s effort, their rank changes (e.g., a rank increase, a rank decrease, no rank change). The user’s effort is a change from a pre-measured baseline of the number of steps and is measured daily. The results are updated and presented daily. In this mechanism, the ease of rank fluctuation is adjusted by the threshold required for rank fluctuation. With a lower threshold, it is easier to obtain results corresponding to efforts in a competition environment. Note that Table 1 (No.1, No.2, No.3) shows a detailed explanation of the amount of the user’s effort, the threshold for a rank fluctuation, and the movement of rivals during rank changes. The rival player is an NPC (non-player character) and behaves based on this mechanism.
**About Score**
  Rivals’ scores change as follows: The score difference between the user and rivals is set by the sum of three factors: the Basic Difference Factor, Effort Factor, and Random Factor. Firstly, the Basic Difference Factor determines the general score difference between the user and rivals. Figure 5 shows an overview of this. For example, by setting Basic Difference Factor to 1000 steps, there is always a rival whose score is different from the user’s score by about 1000 steps. Then, the Effort Factor determines the score difference according to the user’s effort. Figure 6 shows an overview of this. For example, if the user’s effort is large, the difference in the scores compared to higher rivals becomes small. If the user’s effort is small, the opposite occurs. Finally, the Random Factor is used to add randomness to the score difference. In this mechanism, rivals with appropriate score differences can be present in the competition by adjusting the Basic Difference Factor. Note that Table 1 (No.4, No.5, No.6) shows detailed explanations of the Basic Difference Factor, Effort Factor, and Random Factor.

### 3.3. Design of Method 2

The number of rivals to pay attention influences motivation. When using the step log competition information presentation system, the user’s motivation is assumed to be influenced by the number of rivals. For example, the perception of a large number of rivals decreases motivation. A step log competition system is often used at a large-scale competition with many people, such as a health promotion event sponsored by a local government. For example, in Japan, large-scale competition events using a step competition system are held every year in the administrative divisions. Therefore, it is necessary to design a system that prevents the participants from losing motivation due to the presence of many rivals.

**Figure 7:**
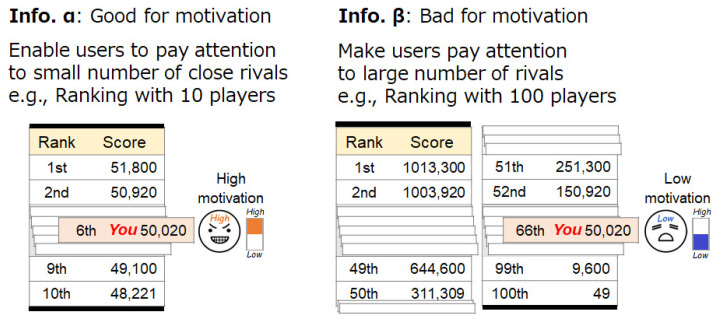
Method to control motivation changes by the number of rivals to pay attention.

**Figure 8:**
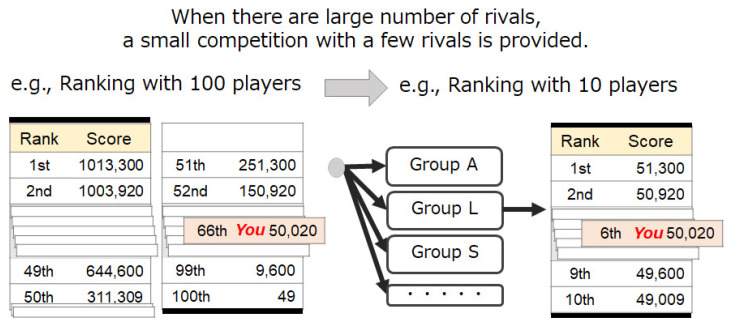
Control of the number of rivals to pay attention.

Therefore, Method 2 has a mechanism that makes a user pay attention to a small number of rivals even when the number of rivals is large. Specifically, Method 2 makes small groups of rivals with similar scores and provides competition information of the small groups. This mechanism also increases the likelihood of being a higher-ranked player in a small group. Figure 7 shows an image of this method. The small groups are made as follows: The default setting of the number of players in a group is 10, as shown in Figure 8. This grouping is updated for every information update. For example, a user in a group who ranks between the 41st and 50th positions is moved to a higher group when his/her rank becomes higher than the 40th position. The user is provided with information about a small-scale group to which he/she belongs and overall competition information.

### 3.4. Prototype System

The system we implemented counts the player’s steps with an activity meter, generates information on a server, and presents the player with this information via a web application that the player can browse with a personal device such as a smartphone. The activity meter was a Pulse O2 from Withings Inc., and the Web application was implemented with JavaScript, PHP, etc.

**Figure 9:**
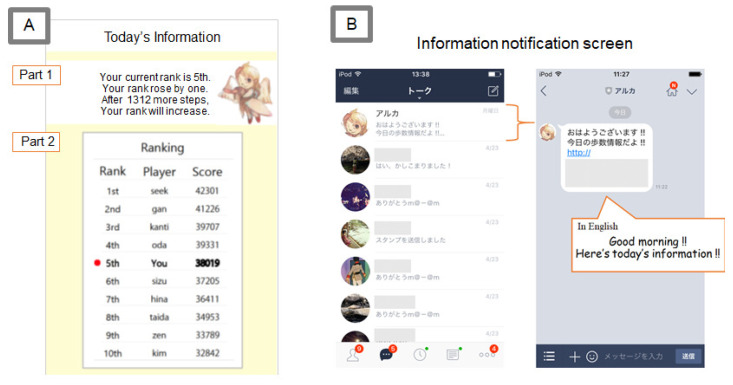
(**A**) Application screen. (**B**) Information notification screen.

The application screen contains two essential parts, as shown in Figure 9A. Part 1 is the comment part, which gives a summary of the essential information by using a character. The aim is to reduce the player’s effort required for interpreting the information and focusing on the primary information. The comments include the player’s current rank, rank fluctuation from the previous day, and the score difference compared to rivals. Part 2 is the ranking part, which provides ranks and scores.

We also implemented a notification application to encourage players to read the competition information with a LINE bot and PHP (LINE is a communication tool from LINE, Inc., Tokyo, Japan). An example of the information notification screen is given in Figure 9B. LINE is a widespread application in Japan commonly used as a daily communication tool that allows users to chat with others, similar to applications such as WhatsApp, Kakao Talk, and WeChat.

## 4. Evaluation

We evaluated our method with 42 subjects. This study was approved by the research ethics committee of Kobe University (Permission number: 29–13) and was carried out according to the committee’s guidelines. The effect of competition information on step counts was measured by dividing subjects into two groups. One group browsed information that is assumed to enhance users’ motivation. The other group browsed information that is assumed to hinder improvements in users’ motivation.

### 4.1. Participants

Participants did not have enough motivation to increase step counts. Detailed explanations are as follows: (1) Age and number of participants:The average age was 22.6 years (standard error: 0.2); 42 university students (38 males and 4 females). (2) Motivation level: Participants did not have enough motivation to increase the number of steps they took. The current stage of motivation was measured with the questionnaire shown in Table 2, which was based on a behavior change stage model (transtheoretical model (TTM)) [63]. Their average level was 2.2 (standard error: 0.1) in Table 2. (3) Duty of browsing: They understood their obligation to read the information every day. (4) Duty of effort: They understood that they had no obligation to make an effort to increase their step counts.

The competition information was updated between 10 am and 12 am and sent to the subjects. They understood their obligation to view the competition information at least once a day. Note that the subjects did not know that the competitor was an NPC. The names of the NPCs are fake nicknames, and the subjects did not know who the NPCs were. The subject did not receive any compensation for this experiment.

### 4.2. Experimental Period

**Figure 10:**
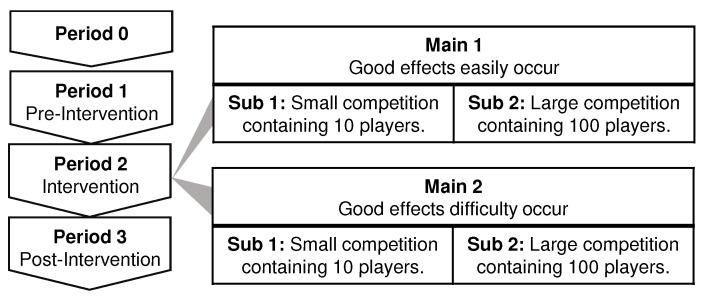
Experimental periods and experimental conditions.

An experimental period consisted of the following four periods, as shown in Figure 10. The experimental period from periods 1 to 3 was six weeks, and each period was two weeks.

- Period 0. Preparation period: Participants had an activity meter for at least two months to get used to carrying a pedometer.
- Period 1. Pre-Intervention: First, a baseline of the number of steps was measured as the average step count during this period. Second, the motivation level was measured with the questionnaire in Table 2. Third, participants were grouped into each experimental condition to eliminate differences in participants’ baseline and motivation levels for each experimental condition.
- Period 2. Intervention: Participants played the game and browsed the presented information every day.
- Period 3. Post-intervention: The competition ended. Participants did not use the application during this period. This period measured the number of steps after the intervention.

### 4.3. Experimental Conditions

The experimental conditions were mainly divided into the following two conditions: Main 1 and Main 2.

**Main 1: A good effect on motivation**
  Main 1 is a condition that is likely to enhance users’ motivation. Main 1 is divided into two groups with different numbers of rivals. A group of Sub 1 were in a competition with ten players. A group of Sub 2 were in a competition with 100 players.
  The characteristics of Main 1 are the following: (1) It is easy to achieve results corresponding to the effort put in. The user’s rank increases and decreases when his/her effort increases or decreases by 1000 steps in a day. (2) There are always rivals within a slight score difference of around 1000 steps. (3) There is information to make subjects pay attention to a few rivals with slight score differences at the time of competition with a large number of rivals. For this, a group of Sub 2 browsed both the the competition information for the condition with ten players and 100 players. A group of Sub 1 browsed the competition information for the condition with ten players.
**Main 2: A bad effect on motivation**
  Main 2 is a condition that is likely to hinder users’ motivation. The same as Main 1, Main 2 also consists of Sub 1 and Sub 2. The characteristics of Main 2 are the following: (1) It is not easy to achieve results corresponding to the effort put in. A user’s rank increases and decreases when his/her effort increases or decreases by 8000 steps in a day. (2) There are always rivals with a large score difference of around 8000 steps. (3) There is no information to make subjects pay attention to a few rivals with slight score differences at the time of competition with a large number of rivals. For this, a group of Sub 2 browsed only the competition information for the condition with 100 players. A group of Sub 1 browsed the competition information for the condition with ten players.

The initial rank and score of participants at the start of the game were roughly unified. Specifically, in Sub 1, the initial rank was set to the sixth position, and the initial score was set to about 35,000 steps, the accumulated value from about six days before. In Sub 2, the initial rank was set to the 66th position, and the initial score was set to about 300,000 steps, the accumulated value from about 50 days before.

## 5. Results and Discussion

**Figure 11:**
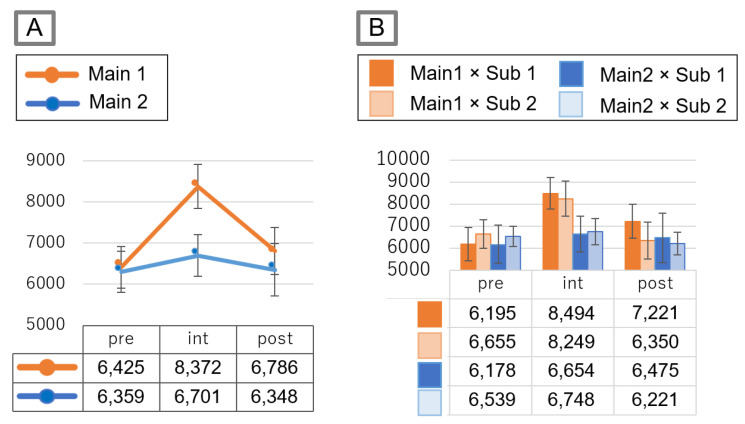
Experimental results. (**A**) Changes in the number of step counts per the Main condition for each period. (**B**) Changes in the number of step counts per the Main condition x the Sub condition for each period.

Figure 11A shows the average step counts for each period. Error bars are standard errors. Figure 11B shows the average step counts for each period under each condition. Error bars are standard errors. A two-way ANOVA and multiple comparisons by Bonferroni correction were used. Periods 1, 2, and 3 are the pre-intervention period, intervention period, and post-intervention period, respectively.

The interaction effect regarding Main × Period was significant trend [F (2,76) = 3.0, *p* < 0.1]. Note that the interaction effect was significant trend, which is not a significant difference considering the Fisher criterion of 0.05. Then, Main at Period 2 was significant [F (1,76) = 4.68, *p* < 0.05], which means that the step counts at Period 2 (i.e., Intervention period) was significantly higher in the Main 1 condition than in the Main 2 condition. Then, Period at Main 1 was also significant [F (2,76) = 9.05, *p*< 0.01] and step counts in Period 2 (i.e., Intervention period) significantly increased compared to those in Period 1 [*p* < 0.05].

### 5.1. Can the Competition Information Be Effective or Not for Improving Motivation?

There was a difference in the number of steps in the intervention period between Main 1 and Main 2. In Main 1, the number of steps during intervention significantly increased than during pre-intervention periods (*p* < 0.05). On the other hand, the number of steps in Main 2 did not increase with the intervention. These results indicate that the effect of competition information on motivation changes depending on the presented information. These results also indicate that a competition information presenting system for supporting improvements in motivation can be effective or ineffective depending on the information pattern.

### 5.2. Can People with Low Levels of Motivation Increase Their Step Counts?

Although the subjects had low levels of motivation, the subjects in Main 1 increased their steps. This result shows that the low-motivated subjects were desirably affected by a competition information presentation designed to improve motivation. Previous studies also showed that subjects with low levels of motivation were unconsciously affected by information presentation designed based on psychological effects and cognitive biases. Similar to previous studies, the effect on subjects in this experiment is considered to be the result of the psychological effect caused by the information presentation.

In addition, the number of steps that could be increased with our system was about 1000 steps. The reason for this was thought to be because there was a rival with a score difference of about 1000 steps, and there was a mechanism causing a rank fluctuation of about 1000 steps. This increase in the number of steps improves health to some extent. For example, the Ministry of Health, Labour and Welfare of Japan has defined an increase in the number of steps from 1000 to 1500 steps per day as suitable for promoting health. This indicates that our information presentation method can increase the number of steps that are meant for health promotion.

Subjects in the group of Sub 1, which has fewer rivals, appear to slightly increase the number of steps compared with subjects in the group of Sub 2, which has many rivals. The N-effect could explain the reason for this. Since it is known that motivation is more likely to increase when the number of rivals is small due to the N-effect, the motivation of subjects in Sub 1 seemed to become higher.

The proposed method did not sustain the increase in the number of steps after the intervention. This means that the effect of the proposed method works while viewing the competition information. Since the proposed method is only designed to affect users only when information is viewed, this result is considered to be reasonable.

### 5.3. Possibility That Competition Information Hinders Improvements in Motivation

The subjects in Main 2 could not increase their number of steps. This result indicates that the information presentation made the subjects maintain low levels of motivation by hindering the subject’s motivation with regard to increasing step counts.

The reason for the slight increase in the number of steps during the intervention period of subjects in Main 2 can be attributed to the fact that they saw the competition information. However, it can be interpreted t hat the increase in the number of steps was not sufficient because the content of the information was not motivating enough.

On the other hand, this experiment could not confirm an apparent bad effect of information on motivation in Main 2, such as a decrease in step counts. Therefore, it can be interpreted that Main 2’s information hinders motivation to improve or was difficult to positively affect motivation. Note that the result of no decrease in the number of steps in the intervention period can be considered reasonable since it is difficult to reduce the number of steps from the usual number of steps in the pre-intervention period.

### 5.4. Design Implication

The experimental results showed that the effect of a competition information presentation on a user’s motivation could be good or not depending on how the information is presented. It also showed that the system side or developers could intentionally cause such an effect. These results include a suggestion for users to carefully use the competition information presentation system and a suggestion for developers to effectively design the competition information presentation system for behavior change support. This section describes each of them.

The experimental results indicated that the use of competition information presentation systems can positively and negatively affect users’ motivation. Understanding this point is necessary when using and providing the competition information presentation system. In particular, it is necessary to take measures against when the competition information presentation system has a negative effect on motivation. For example, to prevent the motivation and effort for target behavior from becoming lower than those before using the system, a mechanism to sense it and tell a user to stop using the system may be helpful. Such a mechanism does not currently exist. In the future, we examine measures for users who cannot get good effects from competition information and users who do not benefit from it.

The experimental results showed the possibility that a competition information presentation system can be made more effective in supporting behavior change. Previous research shows the necessity of designing it in such a way to control the user’s unconscious response (e.g., psychological effects) caused by the information presentation system. By following such a design policy, the competition information presentation system can more effectively support users’ behavior changes than the conventional design by making a mechanism that actively tries to ensure a good effect is achieved from the competition information and suppresses potentially bad effects. One example of this mechanism is the information presentation method designed in this study.

When using the information presentation method we designed in this study, auxiliary players who support users’ motivation are necessary for competition. There are a plurality of patterns to select a person who performs as this auxiliary player. The first is the NPC. NPC means non-player character in computer games [68]. An NPC ideally plays so that a target user can have the intended experience. The second is a real player. In this case, the scores of rivals who are real players are modified to support a user, and the competition information changes for each user. As a point to be noted in this case, the modified information contains some false information. When information that users believe to be true is found to be false, users may lose faith in the system’s credibility. Therefore, it is necessary to make users understand a system design in advance when using this pattern. In addition, there is another pattern without using false information and NPC, such as grouping real players who are prone to positivity, such as those designed in this study (e.g., those who have a similar amount of effort and scores).

Whether or not to use the competition system of such a design must be decided according to the purpose and policy of using competition. There are two types of competition. One is a pure competition that aims to determine the superiority or inferiority of ability with others. For example, sports that professional athletes perform. The other is a competition that has purposes other than pure competition. This kind of competition includes one that aims at behavior change support, such as health and children’s studies. Among these, the latter competition is suitable for using the design of our proposed method.

### 5.5. Abuse of a Competition Information Presentation System to Hinder a User’s Behavior Change

The experimental results indicated that a competition information presentation system can be abused to hinder a user’s behavior change without their awareness. For example, the competition system of step count logs can be used to hinder certain people’s healthy exercise behaviors. Such abuse can be applied to a variety of behaviors in which the competition system is used. For example, it is conceivable that this can not only happen for healthy exercise behavior, but also for learning behavior, working behavior, and volunteer activities. Such abuse can be applied to an organization/country that is inconvenient to another organization/country. Techniques for such abuse are defined as the dark side interface. In addition, deceiving users by presenting information to lead them in the wrong direction is called deception. Even in a competition information presentation system for behavior change support, it is necessary to consider the possibility of such abuse. In the future, we will examine a measure that prevents and detects such abuse.

### 5.6. Limitation

In this verification, there were some limitations, as outlined in the following points. In the future, it is necessary to conduct verification with these considerations.

Since we assumed that the subjects complied with the obligation to view the competition information at least once a day, we did not obtain data on the time and frequency with which the subjects viewed the competition information. Another limitation is the setting where the competition information was updated once a day. At present, there are many step count competition applications that update the number of step counts in real time. If we used an application that updates the number of step counts in real time, the effect of the proposed method may be different from the results of this experiment. Therefore, it is necessary to consider that the results of this study only relate to applications where information is updated only once a day. There is a limitation in the analysis: we cannot identify subjects who intentionally stopped participating in the competition and only measured their steps. Such subjects may be in the experimental group in Main 2 because the competition information in Main 2 was likely to interfere with improving motivation. There is also a limitation in that we could not conduct analysis considering the missing data of the daily step counts. In evaluating such a small case study, missing data due to the subject’s withdrawal may seriously bias the results. Since the age and race of the subjects in this experiment remained within a specific range, we plan to expand this for various users. In addition, we plan to verify the phenomena confirmed in this study for various behaviors to which the competition system is applied. There are many fields this could be used in, such as healthcare, learning, volunteer activities, entertainment, rehabilitation, and sports. Although we used a competition of incremental steps this time, we plan to use further tests using the competition per daily steps in the future because the competition per daily steps is a popular design. In the future, we will have a control group with no interventions to ensure the absence of time effects or effects of prolonged study participation.

## 6. Conclusions

We proposed a method to intentionally control the psychological effect of competition information on behavioral motivation to promote the good effects of competition information systems. We implemented a prototype system using a step count log competition system to support walking motivation. We designed information presentation methods to show ranks, scores, and the number of rivals. We conducted our evaluations over six weeks, involving 42 people who had low levels of motivation to increase their step counts. The results showed that the step counts of subjects changed by about 1000 steps depending on the information patterns with the proposed method. The results showed that the proposed method effectively manipulates the psychological effects of competition information to support behavioral change. In addition, the results showed an example of competition information that is good for supporting behavioral change and not good for it.

## Figures and Tables

**Table 1:** Explanation.

No.1. The amount of user’s effort (EffortAmount): This is defined as $StepAmount_{Oneday}$ − $StepAmount_{baseline}$. $StepAmount_{Oneday}$ is the number of steps during 24 h. $StepAmount_{baseline}$ is a pre-set baseline to measure a user’s effort, and a default setting of it is the average value of the user’s step count during a specific period (e.g., one week before the game).
No.2. Threshold for a rank fluctuation ($Threshold_{RankChange}$): User’s rank changes as an integer part of EffortAmount ÷ $Threshold_{RankChange}$. For example, when EffortAmount > $Threshold_{RankChange}$ × ± *k*, user’s rank changes by ± *k*.
No.3. Rival’s movement during rank changes: Rivals behave as follows to change ranks. Rank-up is provided by moving a rival at one higher rank to one lower level than a user. Rank-down is provided by moving a rival at one lower rank to one higher level than a user. In addition, the following functions work so that rivals are always placed at ranks that can provide rank fluctuations to users. When a user becomes the highest rank (1st.), there are no rivals that can provide the user an event of rank-up. Therefore, if a user is the highest rank (1st.), the following rule is used. (1) The user’s rank remains the highest rank (1st.) when the user’s effort exceeds the rank-up threshold. (2) The user’s rank decreases when the user’s effort does not exceed the threshold for rank up.
No.4. Basic Difference Factor: This determines the basis of the score difference between a user and rivals. It is defined as follows: Rival${}_{n}$ represents a rival whose rank differs from the user’s rank by *n*. Basic Difference Factor for each Rival${}_{n}$ = $BD_{n}$. When $BD_{n}$ sets as 1000 × *n*, the score difference between the user and Rival${}_{n}$ spreads at about 1000 intervals linearly as *n* becomes farther.
No.5. Effort Difference Factor: This factor reflects the user’s effort on a rival’s score. For example, when $Threshold_{RankChange}$ is 1000 steps and EffortAmount is 800 steps, this element makes the user’s score closer to higher rank rivals even though the user’s rank does not rise. This is defined as ($BD_{n}$ × *m*) × (the fractional part of EffortAmount ÷ $Threshold_{RankChange}$ ) and adjusted with *m* (0 < *m* < 1.0). The default setting of *m* is 0.3. Its sign is − × (sign of EffortAmount) × (sign of *n*).
No.6. Random Factor: This factor gives minor randomness to score differences. It is defined as ±($BD_{n}$ × *l*) and adjusted by *l* (0< *l* <1.0). The default setting of *l* is a random value from 0 to 0.2. This was selected as little randomness.

**Table 2:** Questionnaire of motivation level.

1: Pre-contemplation: I do not want to increase my amount of exercise at this time.
2: Contemplation: I would like to increase my amount of exercise.
3: Preparation: I have a plan to change my actions to increase my amount of exercise within the next month.
4: Action: I have changed my behavior as planned and have been doing so for less than six months.
5: Maintenance: I have been able to sustain my change in actions as planned for over six months.
